# Phylogenetic Quantification of Intra-tumour Heterogeneity

**DOI:** 10.1371/journal.pcbi.1003535

**Published:** 2014-04-17

**Authors:** Roland F. Schwarz, Anne Trinh, Botond Sipos, James D. Brenton, Nick Goldman, Florian Markowetz

**Affiliations:** 1 University of Cambridge, Cambridge, United Kingdom; 2 Cancer Research UK Cambridge Institute, Cambridge, United Kingdom; 3 European Molecular Biology Laboratory, European Bioinformatics Institute, Hinxton, United Kingdom; 4 Department of Oncology, University of Cambridge, Cambridge, United Kingdom; ETH Zurich, Switzerland

## Abstract

Intra-tumour genetic heterogeneity is the result of ongoing evolutionary change within each cancer. The expansion of genetically distinct sub-clonal populations may explain the emergence of drug resistance, and if so, would have prognostic and predictive utility. However, methods for objectively quantifying tumour heterogeneity have been missing and are particularly difficult to establish in cancers where predominant copy number variation prevents accurate phylogenetic reconstruction owing to horizontal dependencies caused by long and cascading genomic rearrangements. To address these challenges, we present MEDICC, a method for phylogenetic reconstruction and heterogeneity quantification based on a **M**inimum **E**vent **D**istance for **I**ntra-tumour **C**opy-number **C**omparisons. Using a transducer-based pairwise comparison function, we determine optimal phasing of major and minor alleles, as well as evolutionary distances between samples, and are able to reconstruct ancestral genomes. Rigorous simulations and an extensive clinical study show the power of our method, which outperforms state-of-the-art competitors in reconstruction accuracy, and additionally allows unbiased numerical quantification of tumour heterogeneity. Accurate quantification and evolutionary inference are essential to understand the functional consequences of tumour heterogeneity. The MEDICC algorithms are independent of the experimental techniques used and are applicable to both next-generation sequencing and array CGH data.

## Author Summary

Cancer is a disease of random mutation and selection within the cellular genomes of an organism. As a result, when advanced disease is diagnosed, the cells comprising the tumour show a great amount of variability on the genomic level, a phenomenon termed intra-tumour genetic heterogeneity. Heterogeneity is thought to be one of the main reasons why tumors become resistant to therapy, and thus hinders personalised medicine approaches. If we want to understand tumour heterogeneity and its connection to resistance development we need to quantify it, which implies reconstructing the evolutionary history of cancer within the patient. Unfortunately, so far, methods for accurate reconstructions of these particular evolutionary trees and for quantification of heterogeneity have been missing. We here present MEDICC, a method that uses a minimum evolution criterion to compare cancer genomes based on genomic profiles of DNA content (copy-number profiles). It enables accurate reconstruction of the history of the disease and quantifies heterogeneity. It is specifically designed to deal with diploid human genomes, in that it disentangles genomic events on both parental alleles and includes a variety of accompanying algorithms to test for shapes of the evolutionary trees as well as the rate at which the cancer evolves.

This is a *PLOS Computational Biology* Methods article.

## Introduction

The study of intra-tumour genetic heterogeneity (for short: heterogeneity) is now a major focus of cancer genomics research [1]–[12] due to its potential to provide prognostic information [13]–[15] and to explain mechanisms of drug resistance [16]–[19]. Quantifying tumour heterogeneity and understanding its aetiology crucially depends on our ability to accurately reconstruct the evolutionary history of cancer cells within each patient. In many cancers, such as high-grade serous ovarian cancer (HGSOC), most of this heterogeneity is not reflected in point mutations but in genomic rearrangements and endoreduplications that lead to aberrant copy-number profiles [20], [21]. In these cases tree inference is hindered by unknown phasing of parental alleles and horizontal dependencies between adjacent genomic loci. Therefore heterogeneity and evolutionary divergence are typically quantified using ad-hoc thresholds [19] and tree inference is often done subjectively [11]. Approaches developed to address this problem include a graph theoretical approach on signed reversals to order rearrangement events [22], but this requires detailed annotation of rearrangements in the data that may not be available, and the algorithm does not generally infer global trees representing cancer evolution within a patient. The *TuMult* algorithm [23] deals with underlying computational complexity by considering only breakpoints — locations on the genome where the copy-number changes — and by using total copy-number without phasing of parental alleles. While simplifying the computational problem, this approach discards potentially informative data.

Our aim is to establish numerical quantification of tumour heterogeneity per patient from copy-number profiles that can routinely be acquired from clinical samples. To this end, we have developed MEDICC (Minimum Event Distance for Intra-tumour Copy-number Comparisons), a method for accurate inference of phylogenetic trees from unsigned integer copy-number profiles. MEDICC specifically addresses the following challenges associated with copy-number-based phylogeny estimation:

1. It makes use of the full copy-number information across both parental alleles by *phasing* copy-number variants, i.e. assigning them to one of the two physical alleles such that the overall evolutionary distance is minimal.
2. It estimates evolutionary distances, thereby dealing with *horizontal dependencies* between adjacent genomic loci and with multiple overlapping events by using efficient heuristics. It therefore works on complete copy-number profiles instead of breakpoints which allows the reconstruction of ancestral genomes.
3. It implements statistical tests for molecular clock (homogeneous branch lengths), star topology (phylogenetic structure) and tests for the relationship between clonal subpopulations to provide informative *summary statistics* for the reconstructed evolutionary histories and tumour heterogeneity.

**Figure 1:**
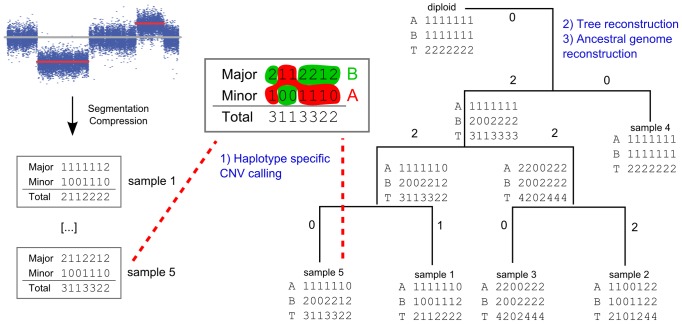
Evolutionary copy-number trees are reconstructed in three steps. 1) After segmentation and compression, major and minor alleles are phased using the minimum event criterion. 2) The tree topology is reconstructed from the pairwise distances between genomes. 3) Reconstruction of ancestral genomes yields the final branch lengths of the tree, which correspond to the number of events between genomes.

**Figure 2:**
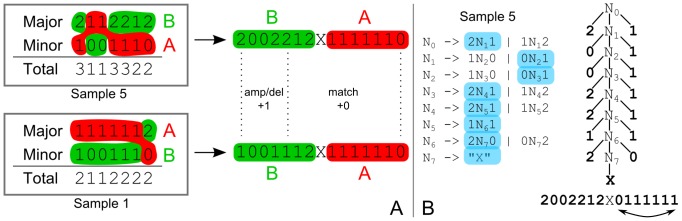
Parental alleles are phased using context-free grammars. A) Allelic phasing is achieved by choosing consecutive segments from either the major or minor allele which minimise the pairwise distance between profiles. B) The set of all possible phasing choices is modelled by a context-free grammar. In this representation, the order of the regions' copy-number values on the second allele is reversed, in order to match the inside-out parsing scheme of CFGs. That way every possible parse tree of the grammar describes one possible phasing.

MEDICC was designed to work on integer copy-number profiles that can routinely be obtained from single nucleotide polymorphism (SNP) arrays [24] or paired-end sequencing [25],[26]. In both cases DNA content is quantified relative to a diploid normal in windows along the genome. SNPs distinguish the two parental alleles via the B-allelic frequency, i.e. the amount of DNA assigned to the B allele relative to the total DNA amount at that specific genomic locus. The resulting profile comprises two vectors of integer copy-numbers, representing the absolute number of copies of that particular genomic segment in the two alleles. However, without any external linkage information these vectors contain no information about which copy-numbers belong together on the same allele [11]. By convention (and for each genomic segment independently), the larger of the two copy-numbers is termed the *major* and the other the *minor* copy-number (Figure 1 left). The process of finding the correct assignment of major and minor copy-number to the two parental alleles is called *phasing*. In contrast to nucleotide substitution models where sites in a sequence are modelled as independent and identically distributed [27], copy-number events often overlap and range across many adjacent genomic regions. Therefore, finding the correct phasing is essential to accurately estimate evolutionary distances (Figure 2A), which additionally requires a model capable of dealing with these horizontal dependencies.

We developed MEDICC and successfully applied it to the analysis of a novel dataset of 170 copy-number profiles of patients undergoing neo-adjuvant chemotherapy for HGSOC as described in our accompanying clinical study [28]. In the following we give a more detailed description of the data and problems that MEDICC addresses. We then introduce the MEDICC modelling framework that guides all steps of the algorithm and which is then explained in detail. We finish with a demonstration of MEDICC on a real-world example of a case of endometrioid cancer and give simulation results that compare it to competing methods.

## Results

Given multiple such evolutionarily-related copy-number profiles, for example from distinct primary and metastatic sites of the same patient, phylogenetic inference in MEDICC then involves three steps: (i) allele-specific assignment of major and minor copy-numbers, (ii) estimation of evolutionary distances between samples followed by tree inference and (iii) reconstruction of ancestral genomes (Figure 1). All three steps are guided by a minimum evolution criterion. Similar to edit-distances for sequence analysis [29], MEDICC counts the number of genomic events needed to transform one copy-number profile into another and searches for the tree that minimises this criterion.

### MEDICC reconstructs evolutionary histories via a minimum evolution criterion

**Figure 3:**
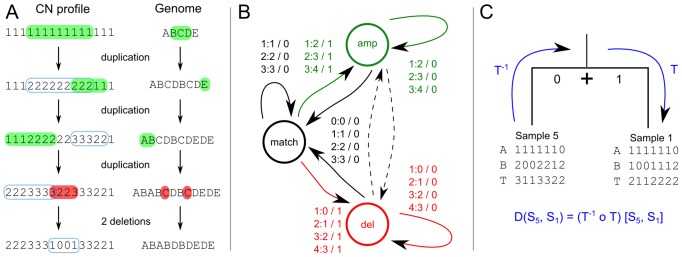
Efficient distance calculation is enabled via a transducer architecture. A) Overlapping genomic rearrangements modify the associated copy-number profiles in different ways. Amplifications are indicated in green, deletions in red. The blue rectangles indicate the previous event. B) The one-step minimum event transducer describes all possible edit operations achievable in one event. This FST is composed 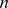 times with itself to create the the full minimum event FST 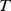. Edge labels consist of an input symbol, a colon and the corresponding output symbol, followed by a slash and the weight associated with taking that transition. C) The minimum event FST 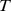 is asymmetric and describes the evolution of a genomic profile from its ancestor. Composed with its inverse this yields the symmetric minimum event distance 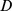.

We model the evolution of copy-number profiles through a series of simple operations that increase or decrease copy-numbers by one (Figure 3A). They map to real genomic rearrangements that have an observable effect on copy-number profiles in the following way: terminal and interstitial deletions, as well as unbalanced translocations, are single deletion events; tandem and inverted duplications are single amplification events; and breakage fusion bridges are dual events involving a duplication and a deletion (copy number decrease on one locus and increase on the second) [22]. We use a finite-state automaton (FSA) representation of genomic profiles and finite-state transducers (FST) [30] for modelling and efficient computing of the minimum-event distance based on these genomic events (Figure 3B). Transducers have earlier been proposed as an efficient way of modeling indels on trees [31]–[33], a problem closely related to the one discussed here. Before going through the three steps of the reconstruction process in detail it is necessary to introduce some terminology; for a more thorough introduction into transducer theory see [30], [34], [35] and references therein.

#### The MEDICC modelling framework

MEDICC models diploid genomic copy-number profiles as sequences over the alphabet 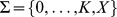, where 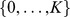 represent integer copy-numbers (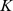 is the maximum haploid copy-number) and 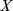 is a special character that separates the two alleles on which events can happen independently. For example, the profile 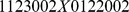 represents a chromosome with 7 regions distinguished, with the first region present in one copy on one allele and absent in the other allele; the second region present in one copy on each allele; and so on up to the seventh region present in two copies on each allele. This means that MEDICC deals with a maximum total copy-number of 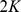 in a diploid genome. By default 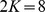 which is the upper end of the dynamic range of SNP arrays, but the alphabet can be extended easily without changing the implementation. In this manuscript the terms “sequence” and “(copy-number) profile” are used interchangeably.

Copy-number profiles are implemented as acceptors, unweighted finite-state automata that can contain a single or multiple such profiles. The minimum-event distance is computed using a weighted finite-state transducer [30]. FSTs are an extension of FSAs with input and output symbols — like pair-HMMs, they emit or accept two sequences simultaneously, meaning they model the events transforming on sequence into another. Both FSAs and FSTs can be equipped with weights from a semiring, enabling calculations to be weighted according to some importance criterion. One of the most common semirings is the real semiring (e.g. the weights represent probabilities), where weights are multiplied along a path in the automaton and the total weight of a sequence (or pair of sequences) is the sum (total probability) over all possible paths generating that sequence. Equally popular is the tropical semiring, also known as the Viterbi path, where weights are summed along a path and the total weight is the minimum across all those paths. In this case weights are often “penalties” or negative log-probabilities for taking a certain path, similar to classical pairwise sequence alignment in which mismatches and indels are penalised with additive fixed scores.

MEDICC uses the tropical semiring for computing the minimum event distance, but the modularity of the framework allows us to smoothly transition to probabilities at a later stage by switching semirings without changing the algorithm. In this tropical semiring a FST 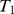 then assigns a score to two sequences (represented as acceptors) 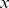 and 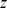 via
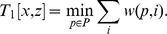
where 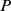 is the set of all possible paths through the FST in which the input and output symbols match with the sequences 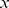 and 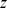 and 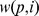 is the weight of that path at position 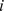 in the sequence. No score is returned for a pair of sequences for which no valid path in 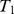 exists. This leads to the definition of the minimum-event distance, which governs all three steps of the reconstruction process.

#### Constructing the minimum-event distance for copy-number profiles

Figure 3B shows the one-step transducer 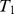 that we use to model single amplifications and deletions of arbitrary length and that counts one event each time the amplification or deletion state is entered. This is analogous to an affine gap cost model in classical sequence alignment [36]. 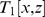 therefore assigns to each pair of sequences 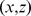 the minimum number of events necessary to transform one sequence into another. At this point, however, not all possible copy-number scenarios have a valid path (e.g. one event can amplify “1” to “2” but not “1” to “3”). To include all possible changes across multiple events, 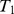 is composed 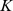 times with itself Mohri2004. In essence, composition describes the chaining of FSTs, where the total weight of the composed transducer is the total minimum score from the input sequence 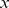 via intermediate sequences 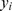 to the target sequence 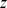:
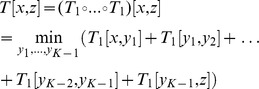
For example, to amplify a copy-number from 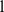 to 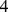 the shortest path goes via two intermediate sequences (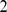 and 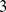) totalling three events (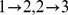 and 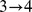).

This composition gives rise to the FST 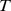 that strictly adheres the modelled biological constraints such as no amplification from zero. We call 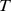 the *tree* transducer: these biological constraints give it a direction, and it is not guaranteed to return a distance for any pair of copy-number profiles. For example, input profile 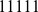 can be transformed into 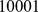 via a single deletion, but not vice versa as once an allele has been lost it cannot be regained.

As we are interested in the minimum evolutionary distance between any two sequences 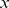 and 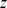 via their last common ancestor (LCA) 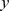, the final distance FST 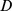 is formed by composing 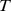 with its inverse (Figure 3C, Schwarz2010), such that 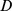 computes the distance from a leaf node to the LCA (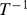) and back (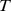) to the other leaf node:
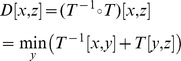
In the real semiring, and equipped with probabilities, this would be analogous to classical phylogenetic reconstructions where a reversible model of sequence evolution is used to compute the likelihood of the subtree containing sequences 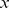 and 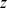 as the products of the individual likelihoods of seeing 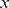 and 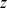 given their ancestor 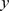 and summing over all 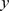 [37]. In our case, 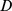 equivalently computes the minimum number of events from 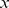 to 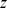 via their LCA. This distance is symmetric and is guaranteed to yield a valid distance for any pair of sequences. In the rest of the paper, “distance” refers to this minimum-event distance, unless stated otherwise.

MEDICC therefore computes an evolutionary distance between two genomes based on a minimum evolution criterion via their closest possible LCA. Due to composition of the tree transducer 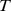 with its inverse, the resulting distance 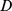 is a dissimilarity score that at the same time is also (the logarithm of) the shortest-path approximation to a positive-semidefinite kernel score [38], [33]. That means that the pointwise exponential of the estimated distance matrix 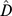, 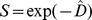, is a positive-semidefinite similarity matrix (with all eigenvalues 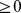). The entries of this matrix are the values of the pairwise dot products of the sample genomes in a high-dimensional feature space. This feature space can be thought of as a space where every possible copy-number profile defines one dimension and sample genome 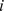 is represented by a numerical feature vector 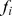 that contains an evolutionary similarity score between the sample genome itself and each of these reference profiles. The entries of the kernel matrix 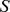 are then simply the dot products 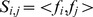 of the feature vectors 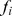 and 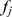. We term this space the mutational landscape in which spatial distances correspond to evolutionary distances and on which we can directly apply explorative analyses like PCA, classification with support-vector machines and other machine learning techniques [39]. We use *OpenFST*, an efficient implementation of transducer algorithms [40] to achieve exact distance computation in quadratic time.

Following the minimum evolution principle, the overall objective is to find a tree topology including ancestral states that minimises the total tree length, i.e. the total number of genomic events along the tree. In the following we will describe how MEDICC achieves this in its three step process.

#### Step 1: Evolutionary phasing of major and minor copy-numbers

As copy-number-changing events can independently occur on either or both of the parental alleles the phasing of major and minor copy-numbers heavily influences the minimum tree length objective. We use the evolutionary information between samples to solve these ambiguities. Using our distance measure we can choose a phasing between a pair of diploid profiles that minimises the pairwise distance between them (Figure 2A). This respects the distinct evolutionary histories of both alleles and finds a phasing scenario in which the evolutionary trajectories between both haploid pairs are minimal. From each pair of major and minor input sequences we can generate up to 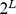 possible phasing choices, where 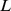 is the length of the input profile (both alleles have the same length). This is too many to evaluate exhaustively, so in order to achieve a compact representation of diploid profiles we make use of a context-free grammar (CFG). Our implementation is related to the use of CFGs to model RNA structures, where paired residues in stem regions are not independent [36].

In our copy-number scenario a CFG represents different allele phasing choices (see Figure 2B right). At every position in the diploid profile we have a choice of using the major as the first allele and the minor as the second or (“
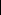
”) vice versa (Figure 2B left). Each possible parse tree of the CFG then corresponds to one phasing scenario out of the 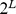 possibilities. When the distance FST reads the separator it is forced to return to the match state (initial state), thus guaranteeing that the total distance to another profile equals the sum of the distances of the two alleles with no events spanning different alleles. We represent CFGs algorithmically by pushdown-automata in the FST library [41].

While this approach works well for finding phasing scenarios that minimise the distance between one pair of profiles, we aim to find phasing scenarios that jointly minimise the distances between all profiles in the dataset. To reduce the computational complexity of this task we have found it necessary to employ a heuristic. MEDICC searches for the single profile that has minimum sum of distances to all sample profiles, that is, the geometric median, through an iterative search. This profile is then compared again to each individual profile and the shortest path algorithm yields the choice of phasing that minimises the distance between each profile and the centre. This approach is not guaranteed to return a globally optimal phasing scenario, but has proven to perform very well in simulations (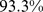 correctly phased genomic loci; see simulation section).

#### Step 2: Distances and tree reconstruction

Once the alleles have been phased, pairwise evolutionary distances between samples can be computed as the sum of the pairwise distances between both alleles. MEDICC then uses the Fitch-Margoliash algorithm [42] for tree inference from a distance matrix with or without clock assumption. A test of clock-like events, available using functionality in the accompanying R package *MEDICCquant*, allows us to determine which tree reconstruction algorithm is most appropriate (see the section on quantification of heterogeneity).

#### Step 3: Ancestral reconstruction and branch lengths

From this point on we keep the topology of the tree fixed, and traverse from its leaves to the root to infer ancestral copy-number profiles and branch lengths. Ancestral reconstruction is possible because cancer trees are naturally rooted by the diploid normal from which the disease evolved. Reconstructing ancestral genomes allows us to investigate e.g. the genomic makeup of the cancer precursor, the LCA of all cancer samples in the patient. Events that across patients frequently occur between the root of the tree and the precursor are likely driver events of cancer progression. Ancestral reconstruction also determines the final branch lengths of the tree. MEDICC infers ancestral genomes for each allele independently using a variant of Felsenstein's Pruning algorithm [27].

In Felsenstein's original algorithm the total score (likelihood/parsimony score) of the tree is computed in a downward pass towards the root and ancestral states are then fixed in a second upward pass, successively choosing the most likely/most parsimonious states. In our scenario, the algorithm begins by composing each of the 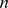 terminal nodes with the tree transducer 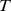, which yields 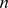 acceptors holding all sequences reachable from that terminal node and their respective distances. When moving up the tree to the LCA of the first two terminal nodes the corresponding acceptors are intersected. The resulting acceptor contains only those profiles that were contained in both input acceptors and their corresponding weights are set equal to the sum of the weights of the profiles in the input acceptors. In a probabilistic framework the resulting acceptor is equivalent to the conditional probability distribution 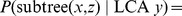 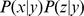 for each possible LCA, where the sum of distances again is replaced by the product of the conditional probabilities of seeing a leaf node given its ancestor. This intersection will still contain the vast majority of all possible profiles, but each with a different total distance, and without those that are prohibited by biological constraints. For example, the ancestor cannot have a copy-number of zero at a position where any of its leaf nodes has copy-number 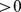, as amplifications from zero are not allowed. Because after phasing each leaf node is represented by an acceptor containing exactly one diploid sequence, computing this set of possible ancestors is computationally feasible. However, because during tree traversal we need to compose these sets of possible profiles repeatedly with the tree transducer 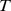, the result would increase in size exponentially because it has to account for all possible events of arbitrary length at each position in all sequences. Therefore during tree traversal, when two internal nodes have to be joined in their LCA, MEDICC reduces each of them to a single sequence by choosing those two sequences with smallest distance to each other. This fixes the profiles for those two internal nodes. This procedure is continued until all internal nodes are resolved. Once all ancestral copy-number profiles have been reconstructed the final branch lengths are simply the distances between the nodes defining that branch in the tree.

### MEDICC improves phylogenetic reconstruction accuracy

We assessed reconstruction accuracy using simulated data generated by the *SimCopy* R package [43] (see Methods). Random coalescent trees were generated with *APE* [44]. To create an unbiased simulation scenario, genome evolution was simulated using increasing evolutionary rates on the sequence level using five basic genomic rearrangement events: deletion, duplication, inverted duplication, inversion and translocation (for details see Methods). Once the simulations were complete, copy-numbers were counted for each genomic segment and these copy-number profiles were used for tree inference using the following three methods: i) BioNJ [45] tree reconstruction on a matrix of Euclidean distances computed directly on the copy-numbers, ii) breakpoint-based tree-inference using the *TuMult* software [23] and iii) MEDICC. *TuMult* additionally requires array log-intensities as input. In order to keep the comparisons unbiased, noiseless log ratios simulating CGH array intensities for *TuMult* were directly computed from the copy-number profiles. To assess the relative abilities of the methods to correctly recover the evolutionary relationships of the simulated copy-number profiles, reconstruction accuracy was measured in quartet distance [46] between the true and the reconstructed tree. Quartet distance was chosen as it only considers topological differences; branch lengths have widely different meanings in the methods tested and as such are not comparable.

This simulation strategy is based on basic biological principles, independent of the methods compared and *a priori* does not favour any of them. All simulations were repeated to cover a wide parameter range, yielding qualitatively similar results.

**Figure 4:**
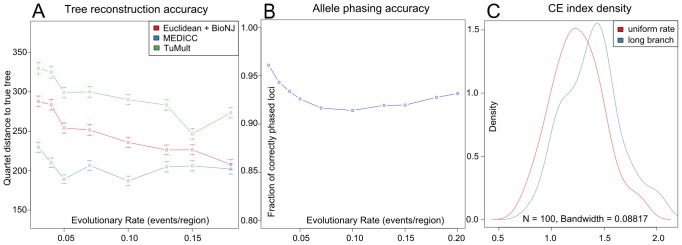
MEDICC improves reconstruction accuracy over competing methods. A) Simulations results show the improvement of reconstruction accuracy for MEDICC over naive methods (BioNJ clustering on Euclidean distances between copy-number profiles, red) and competing algorithms (TuMult, green). B) Allele phasing accuracy across the simulated trees. On average 92.9% of all genomic loci were correctly assigned to the individual parental alleles. C) Density estimates of clonal expansion indices for neutrally evolving trees (red) and trees with induced long branches as created by clonal expansion processes (blue) show the ability of MEDICC to detect clonal expansion.

The simulation results clearly show the improvement in reconstruction accuracy of MEDICC over naive approaches (BioNJ on Euclidean distances) and competing methods (TuMult) (Figure 4A). In general, reconstruction accuracies increase with increasing evolutionary rates. Especially when the amount of phylogenetic information is limited, MEDICC outperforms other methods by a significant margin. This may be because of two reasons: firstly, in contrast to other methods MEDICC is capable of phasing the parental alleles, thereby making much more effective use of the phylogenetic information compared to methods that work on total copy-number alone. Secondly, due to efficient and accurate heuristics, MEDICC can deal with the horizontal dependencies imposed by overlapping genomic events of arbitrary size and accurately computes distances between them.

To assess the accuracy of the implemented CFG-based phasing method, for each reconstructed tree phased alleles were compared to the original simulated alleles. MEDICC correctly phased 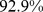 of all genomic loci across all simulations (Figure 4C). We additionally evaluated the runtime of the complete algorithm on our simulation scenario which consisted of 100 genomic segments after compression and found it to take on average 5 minutes for a full reconstruction on a UNIX based Intel(R) Xeon(R) CPU E5-2670 at 2.60 GHz.

### Evolutionary comparisons with MEDICC allow quantification of tumour heterogeneity

Intra-tumour heterogeneity is a loose concept that describes the amount of genomic difference between multiple cells or samples of the same tumour. Two types of heterogeneity often of interest are *spatial* and *temporal* heterogeneity. For example, spatial differences might be observed from separate biopsies of a primary cancer and a distant metastasis. Other changes may occur between different time points, for example before and after chemotherapy. Average distances between subsets of samples might be computed by any method that returns dissimilarities between samples by simple averaging. However, clinical datasets are often noisy due to normal contamination and immune response such as leukocyte infiltration. As for example a sample with exceptionally low cellularity can lead to errors during segmentation, more robust measures of distances between aggregated subsets of samples are desirable that are not easily skewed by outliers.

As described earlier, the matrix of pairwise minimum-event distances inferred by MEDICC can directly be transformed into a kernel matrix [38], [33] which maps samples to a high-dimensional mutational landscape. We reduce the dimensionality of this landscape through kernel principal components analysis [47] where we can use spatial statistics to derive numerical measures of heterogeneity for each patient.

#### Temporal heterogeneity

**Figure 5:**
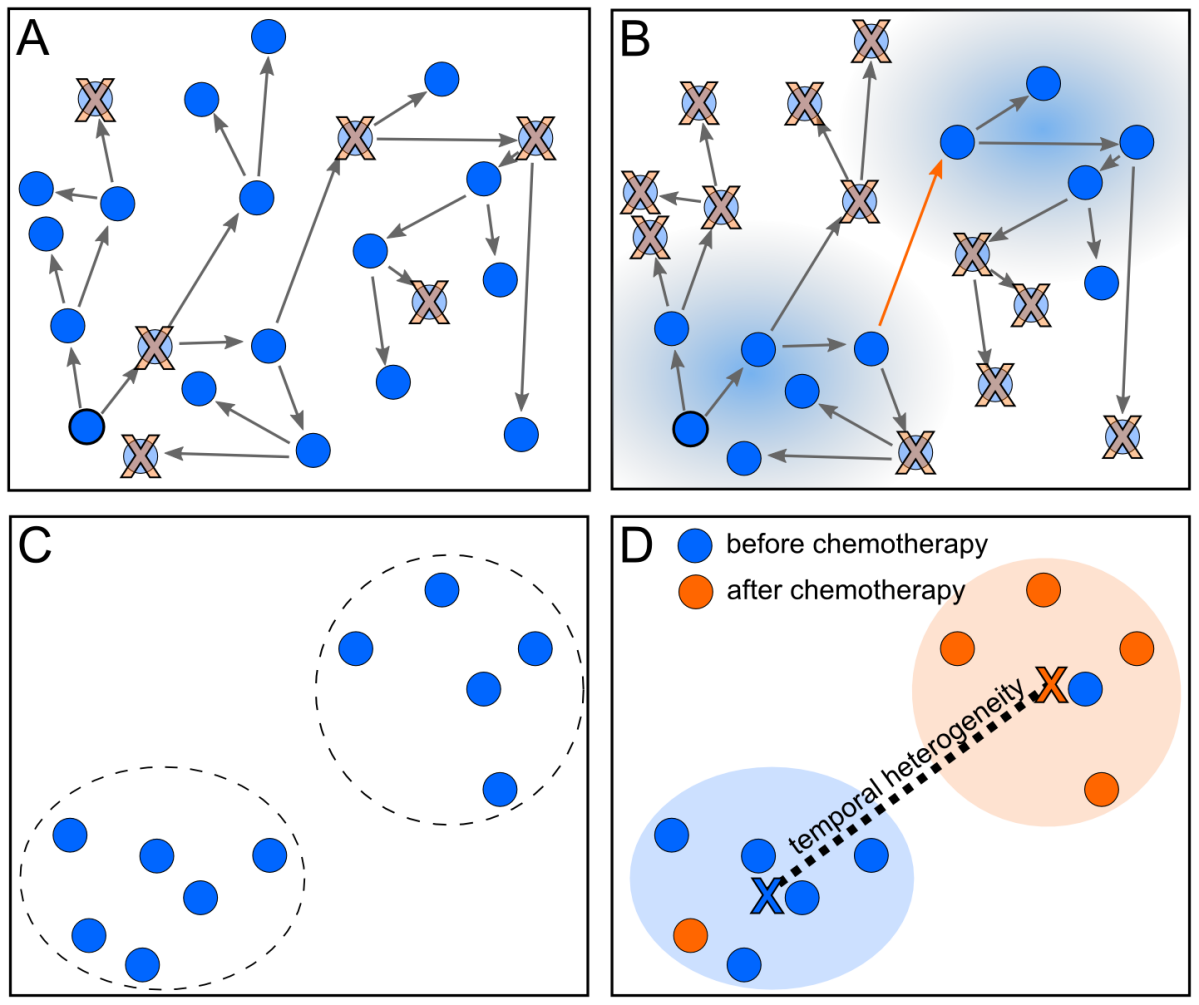
MEDICC quantifies heterogeneity from the locations of genomes on the mutational landscape. A) If no or a homogeneous selection pressure is applied, cells proliferate and die randomly across the mutational landscape, leaving the surviving cells spatially unclustered. B) If the fitness landscape favours specific mutations (blue shaded areas), genomes inside those areas are more likely to survive, those outside more likely to die. The ability of a tumour for a clonal expansion into distant fitness pockets depends on its mutation potential per generation (long orange arrow). This leads to C) a situation where distinct subpopulations/clonal expansions are present in a tumour, indicating a generally high potential for a tumour to adapt to changing environments. D) The mutational landscape additionally allows estimates of average distance between two subgroups of samples, here before (blue) and after (orange) chemotherapy. The distance between the two subgroups is defined as the distance of the robust centres of mass (blue and orange X). This robust centre of mass is computed omitting the single most distant point of each subgroup (blue and orange samples in the orange and blue subgroups respectively), making the statistic more resistant towards outliers.

We define temporal heterogeneity as the evolutionary distance between the average genomic profiles between any two time points (e.g. at biopsy before chemotherapy and at surgery after chemotherapy in the case of neo-adjuvant treatment). In the mutational landscape (see above) we are able to directly compute the centre of mass of a set of genomic profiles (which would not be possible by working with distances alone) (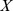 in Figure 5D). The center of mass 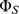 of a set of points 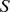 in feature space is defined as
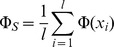
where 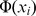 is the feature space mapping of point 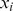 and 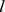 is the number of points. We can then define temporal heterogeneity as the distance between the centres of mass of the samples from two time points. Consider the blue and orange sets in Figure 5D, named 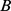 and 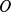 with 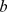 and 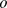 elements respectively. Without loss of generality we can assume our genomic profiles 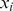 to be partitioned into the two sets such that 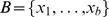 and 
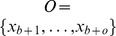
. The squared distance between the centers of mass 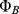 and 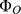 of the two sets of genomic profiles in our feature space, is then defined as:
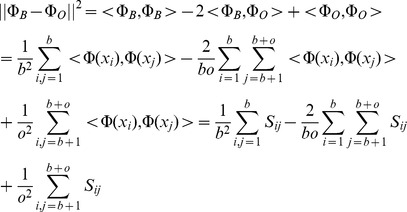
where 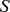 again is the kernel or similarity matrix. An advantage of this approach is that it is possible to replace 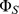 with other robust measures of the centre of mass (e.g. ignoring the single most distant point). It should be noted that this general approach can be used for determining distances between any partitions of the samples in the dataset, for example between groups of samples taken from different organs as a measure of spatial heterogeneity.

#### The clonal expansion index

Other complex aspects of heterogeneity that cannot be easily derived from distances alone include the ability of a tumour to undergo clonal expansions [16]. The model here is that if the majority of cancer cells are subject to strong selection pressure, such as from chemotherapy, minor subclones with a distinctive selective advantage may repopulate. This subpopulation would be expected to coalesce early and will show a greater than expected divergence (relative to neutral evolution) from other remaining clones. This model is similar to analyses of clonality in bacterial populations [48]. Traditional tests for deviation from a neutral coalescent are typically based on single polymorphic sites and often require information about the number of generations [49]. As such information is not available for clinical cancer studies, we therefore make a spatial argument about clonal expansions. We assume that due to the large population sizes of cancer cells, genetic drift is not significant. In a setting of neutral evolution where all sequences have essentially the same fitness, sequences randomly move across the mutational landscape leading to a uniform distribution of sequences in that space (Figure 5A) with no selective sweeps or clonal expansions. If strong selective pressure favours specific mutations (Figure 5B), sequences are more likely to survive and be sampled from the favoured regions leading to local clustering of sequences on the mutational landscape (Figure 5C).

Besag's 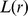 [50], a variance-stabilised transformation of Ripley's 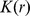 [51], is a function used in spatial statistics to test for non-homogeneity, i.e. spatial clustering, of points in a plane. 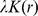 describes the expected number of additional random points within a distance 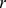 of a typical random point of an underlying Poisson point process with intensity 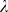. The empirical estimate of Ripley's 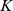 for 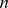 points with pairwise distances 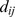 and average density 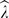 is defined as
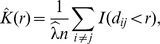
where 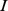 is the indicator function. In case of complete spatial randomness (CSR), the expectation of 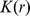 is 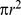. Besag's 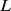 is defined as 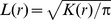 and under CSR has expectation linear in 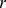. Therefore plotting 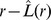 can be used as a graphical indication of deviation from CSR. We use a simulation approach to estimate significance bands for 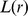 [52].

The clonal expansion index CE for a dataset (typically samples taken from a single patient) is then defined as the maximum ratio between the distance of the observed L-value () and the theoretical L-value under CSR (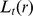) and one-half the width of the two-sided simulated significance band 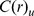 (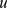 for upper significance band):
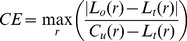
(1)


A value of CE 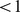 therefore suggests CSR in the point set, whereas a CE value 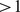 indicates local spatial clustering. We conducted coalescence simulations to confirm that the clonal expansion index distinguishes between trees with normal and elongated branch lengths between populations (black and red distributions, Figure 4B).

#### Testing for star topology and molecular clock

Tree reconstruction methods may or may not include assumption of a molecular clock, and this may significantly influence the reconstruction accuracy. It is of particular interest in cancer biology whether evolution is governed by constant or changing rates of evolutionary change. Furthermore, it is still debated whether disease progression follows a (structured) tree-like pattern of evolution or if subpopulations are emitted in radial (star-like) fashion from a small population of stem-like progenitors (see [53]).

We implemented tests for tree-likeness and molecular clock in the *MEDICCquant* package to help answer these questions. We model genomic events 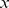 as generated from a Poisson process 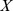 with rate 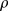. The expected number of events is then linear in time: 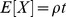. Assuming 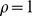, where the process is not time-calibrated, the observed distance 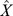 is the maximum likelihood estimate (MLE) for the time of divergence. Under asymptotic normality of the MLE we have that 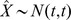. Given a star topology we find optimal branch lengths that minimise the residual sum of squares between the optimised pairwise distances 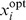 and the measured pairwise distances 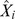 for branch 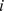. Under the null hypothesis of star-like evolution this sum of squares
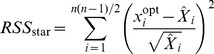
is then 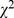-distributed with 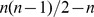 degrees of freedom, where 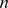 is the number of samples studied, i.e. the number of leaves in the tree. The degrees of freedom is derived from the difference between the numbers of freely estimated distances under the alternative hypothesis (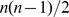 pairwise distances among the 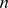 samples) and the null hypothesis (one for each of the 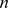 branches in the star topology).

An analogous procedure can be used for testing whether a tree follows a molecular clock hypothesis, in which it exhibits constant evolutionary rates along all branches. In this case the distances 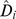 of all leaf nodes from the diploid should be the same. We measure the deviation of the 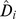 from their mean (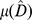) by
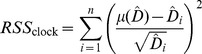



Because branch lengths do not need to be optimised to a specific topology, and we are only considering distances to the diploid, the distribution in this case has 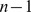 degrees of freedom (the difference between 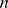 such distances free to vary with no clock, and one distance when there is a molecular clock).

### Progression and heterogeneity in a case of metastatic endometrioid adenocarcinoma

In the following section we demonstrate MEDICC on a case from the CTCR-OV03 clinical study [54]. This case had advanced endometrioid ovarian carcinoma and was treated with platinum-based neoadjuvant chemotherapy. After three cycles of chemotherapy the patient had stable disease based on RECIST assessment, pre- and post-chemotherapy CT imaging and a 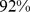 reduction of the tumour response marker CA125. She then underwent interval debulking surgery but had residual tumour of 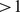 cm at completion. After six moths she progressed with platinum-resistant disease and died one month later.

Out of 20 biopsy samples 18 satisfied quality control for 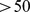 tumour cellularity and array quality. The dataset included 14 omentum samples, two samples from the vaginal vault (VV) and two samples from the external surface of the bladder (BL). The BL and VV samples were taken prior to chemotherapy and the omental samples were collected at interval-debulking surgery after three cycles of chemotherapy.

**Figure 6:**
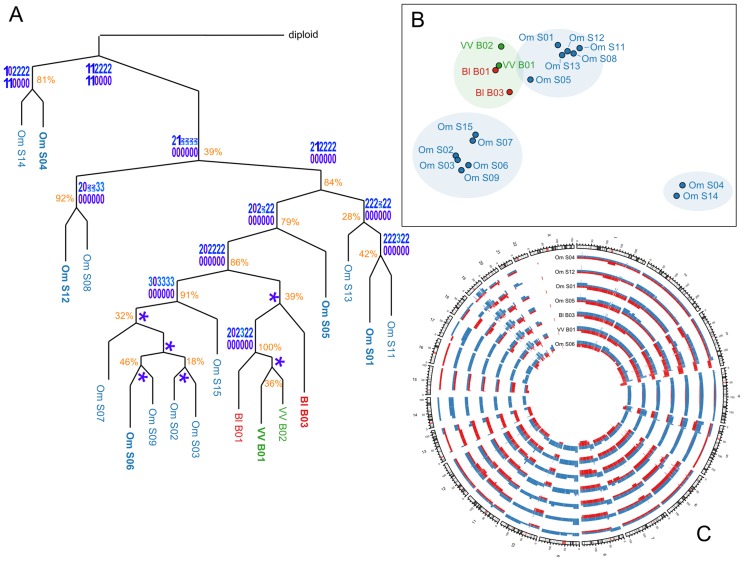
Application to a case of endometrioid cancer. A) Evolutionary tree of the OV03-04 case reconstructed from whole genome copy-number profiles. Approximate support values indicate how often each split was observed in trees reconstructed after resampling of the distance matrix with added truncated Gaussian noise. MEDICC performs reconstruction of ancestral copy-number profiles. Here, the (compressed) ancestral profiles for chromosome 17 are given as an example and MEDICC depicts unresolved ambiguities in the form of sequence logos. A star indicates no change compared to its ancestor. B) Ordination of the samples using kPCA shows four clear clonal expansions, comprising three separate Omentum groups and the Bl/VV group. C) Circos plot of selected genomic profiles (marked in bold in the tree) shows the extent of chromosomal aberrations across the genome. The two phased parental alleles are indicated in red and blue.

All samples were copy-number profiled with Affymetrix SNP 6.0 arrays and segmented and compressed using PICNIC [24] and CGHregions [55]. Pairwise evolutionary distances between all samples were estimated with MEDICC. The distance distribution was tested for the molecular clock hypothesis using MEDICCquant and showed strong non-clock like behaviour (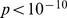, Figure 6A). Tree reconstruction was performed by MEDICC using the Fitch-Margoliash algorithm Fitch1967. MEDICCquant detected a high degree of clonal expansion (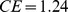) as can be seen in the strong spatial clustering of samples on the mutational landscape (Figure 6B). MEDICC counted a median of 204 genomic events relative to the diploid and a median of 146 between all pairwise comparisons. Tree reconstruction showed good support values for the omental and BL/VV subclades, suggesting strong spatial heterogeneity. The patient also showed strong temporal heterogeneity, as there were large evolutionary distances between samples before and after neoadjuvant chemotherapy (temporal heterogeneity index 3.78, Figure 6B). However, temporal and spatial heterogeneity in this case are indistinguishable because the BL/VV samples coincide with the biopsy samples, whereas all omentum samples were taken at surgery.

Ancestral reconstructions using MEDICC showed loss-of-heterozygosity (LOH) events on chromosome 17q (see internal node profiles in Figure 6A) that often coincide with deleterious mutations in BRCA1 and TP53 [56]. The most prominent contributors to the clonal expansions of the subgroup surrounding sample S01 seemed to be chromosomal amplifications on chromosomes 6, 8, 11 and 14; as well as LOH on chromosome 15. We also detected large LOH events on chromosomes 4, 5, 9, 10, 13, 14, 16 and 17 (Figure 6C).

## Discussion

While significant progress has been made recently to understand tumour heterogeneity through extensive multiple sampling studies and experimental efforts, few algorithms have been developed to target the specific questions raised by such datasets. MEDICC is our contribution to better reconstruct the evolutionary histories of cancer within a patient and propose unbiased quantification of heterogeneity and the degree of clonal expansion.

We have shown the success of these efforts in simulations and their utility in the example discussed in this article. More detailed analyses of clinical cases that also elaborate on the connection between clonal expansions and patient outcome can be found in our clinical study [28].

It is important to note that both the clonal expansion index and the proposed measure for average evolutionary distance between subsets of samples are based solely on pairwise distances and the implicit feature space projection and not on the reconstructed trees. This is advantageous as e.g. for the temporal heterogeneity index the subsets of samples that are compared are not necessarily monophyletic clades in the tree.

As discussed above we attribute the increase in reconstruction accuracy mainly to two factors. First, MEDICC makes efficient use of the available phylogenetic information by phasing parental alleles using the minimum evolution criterion, which has to our knowledge not been attempted before. Second, MEDICC models actual genomic events that change copy-number and incorporates biological constraints such as loss-of heterozygosity, which is not the case in breakpoint-based approaches.

The loss of reconstruction accuracy of *TuMult* relative even to naive approaches using Euclidean distances is most likely due to the fact that *TuMult* was designed for fewer leaf nodes (typically around 4; *Letouze, personal communication*). It is worth stressing that, unlike its competitors, MEDICC is not linked to a specific data collection platform. Data from SNP arrays can be used, as well as sequencing-based datasets or any other method that returns absolute copy numbers. It is further worth noting that an increase in 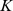, the maximum allelic copy-number, first of all increases the alphabet size but not the complexity of the algorithms. However, increasing 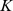 also increase the number of states in the tree FST 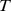 and hence the memory demands on the elementary FST operations *determinisation* and *minimisation* [34] that are used when constructing 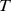. This effectively caps 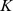 at a value of 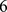 for the time being.

Future work will focus on reductions of algorithmic complexity as well as the integration of SNV data into the reconstruction process. Another important aspect is subclonality within a physical sample which may not easily lead to integer-based copy number inference. Instead of fully clonal integer CN profiles we are working on an extension that allows for mixtures of cells to be represented effectively, allowing for the computation of expected sequence similarities between mixtures of cancer genomes.

Additionally, it would certainly be desirable to move from the current minimum-evolution approach to a full probabilistic model with specific probabilities for amplification and deletion events. Event probabilities could then be trained by expectation-maximisation. However, this significantly increases the computational complexity of the algorithm, which demands the development of new heuristics that constrain the size of intermediate results of the reconstruction process.

Another consequence of this minimum-evolution approach is that all events are weighted equally, independent of their size, while computing evolutionary distances. During ancestral reconstruction, however, if two possible ancestors would yield the same total number of events in the tree, the algorithm prefers shorter events over longer ones to reduce the ambiguity when determining ancestral genomes. Preferring shorter events is a direct consequence of our minimum evolution approach. However, if two genomes differ by a focal deletion in a key gene that confers a substantial fitness advantage, this fitness-increasing mutation will most likely not be visible when determining the clonal expansion index due to its relative small evolutionary distance to the other genomes. Future work might explore the possibility of weighting individual events based on their genomic position and the potential oncogenes and tumour suppressors contained therein.

Lastly, MEDICC is subject to the same limitations as classical algorithms for phylogenetic reconstructions. Strong convergent evolution, i.e. two genomes becoming similar due to selection even though they diverged early, can in theory mislead the reconstruction process. However, this problem is typically more pronounced for point mutations than for copy-number changes. Two convergent copy-number events that occurred independently must by chance have the same start and end locus on the genome to be considered identical, which is much less likely than two point mutations occurring by chance at the same genomic position, due to the far greater number of possible outcomes of each event.

## Methods

SNP array data for the example from the OV03/04 study can be accessed at the NCBI Gene Expression Omnibus under accession number *GSE40546*.

### Simulation of tumour evolution

Coalescent trees were simulated using the *APE* R package [44]. Simulation of genome evolution on these trees was performed by custom code, released as the *SimCopy* R package [43]. *SimCopy* relies on the *PhyloSim* package [57] in order to perform the simulations on the level of abstract “genomic regions”. The genomic regions are encoded in a sequence of integers, with the sign representing their orientation. The package then uses modified *PhyloSim* processes in order to simulate deletion, duplication, inversion, inverted duplication and translocation events happening with rates specified by the user. The number of genomic regions affected by each of these events is modelled by truncated Geometric+1 distributions. After simulating genome evolution, copy-number profiles are reported for leaf and internal nodes. Genomes were simulated using 15 leaf nodes, a root size of 100 segments and an average event length of 12 segments to allow for overlapping events. Event rates covered the following set: 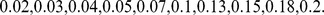 Individual event rates were modified with the following factors: deletions: 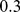, duplications: 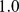, inverted duplications: 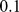, inversions: 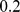, translocations: 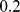. All parameters were chosen such that the leaf node copy-number distributions are similar in shape to copy-number distributions from experimental data in the clinical study [28].

### Implementation of MEDICC

All FST and FSA algorithms were implemented using OpenFST [40]. MEDICC was written in Python, while implementation of time-critical parts used C. For the Fitch-Margoliash implementations we used the Phylip package [58]. MEDICC is available at https://bitbucket.org/rfs/medicc and has been tested on Windows and UNIX-based systems.

The quantitative analysis of MEDICC results was done in R and all necessary functions are implemented in the *MEDICCquant* package included in the MEDICC distribution. Spatial statistics were computed using the *spatstat* package [52], and for kernel manipulations the *kernlab* package was used [59].
